# A systematic review of symptom assessment scales in children with cancer

**DOI:** 10.1186/1471-2407-12-430

**Published:** 2012-09-26

**Authors:** L Lee Dupuis, Marie-Chantal Ethier, Deborah Tomlinson, Tanya Hesser, Lillian Sung

**Affiliations:** 1 Department of Pharmacy, The Hospital for Sick Children, 555 University Avenue, Toronto, ON, M5G 1X8, Canada; 2 Program in Child Health Evaluative Sciences, The Hospital for Sick Children, 555 University Avenue, Toronto, ON, M5G 1X8, Canada; 3 Division of Haematology/Oncology, The Hospital for Sick Children, 555 University Avenue, Toronto, ON, M5G 1X8, Canada; 4 Leslie Dan Faculty of Pharmacy, University of Toronto, Toronto, Canada

**Keywords:** Symptom, Screening, Assessment, Scale, Children, Cancer

## Abstract

**Background:**

The objective was to describe symptom assessment scales that have been used in children with cancer.

**Methods:**

We conducted electronic searches of OVID Medline and EMBASE in order to identify all symptom assessment scales that have been used in pediatric cancer. Two reviewers abstracted information from each identified study. Data collected included study demographics and information related to the instrument and children enrolled. We also collected information about the purpose of instrument administration and whether treatment was altered as a result of this information.

**Results:**

Fourteen studies were identified which evaluated eight different symptom assessment scales. Eight studies used child self-report and all studies included children on active treatment for cancer although 4 studies also included children following completion of treatment. The most common purpose of instrument administration was to measure the prevalence of symptom burden (n = 8). None of the 14 studies used the scale to screen for symptoms and none changed patient management on the basis of identified symptoms.

**Conclusions:**

We failed to identify any symptom assessment scales that were used as a symptom screening tool. There is a need to develop such a tool for use in children with cancer.

## Background

Cure rates for pediatric cancer are approaching 80% but the costs of this progress include a high prevalence of symptoms during treatment [1-3] and a high rate of chronic health conditions following completion of treatment [4]. It is important to identify and control symptoms in order to maximize quality of life (QoL) and reduce morbidity. Furthermore, there is some evidence that reduction in symptoms may improve future psychosocial functioning [5].

Within the adult oncology setting, screening of symptoms through patient self-report has been identified as an important priority [6-9]. Consequently, much effort has been focused on symptom screening and control. In particular, efforts by Cancer Care Ontario have culminated in the wide-spread use of a symptom screening tool based upon the Edmonton Symptom Assessment Scale (ESAS) [10]. The ESAS is a validated symptom screening tool which asks adult patients to rate the severity of nine common symptoms including pain, anxiety and nausea. In a satisfaction survey conducted in 2010 among 2,921 patients, 87% of respondents thought that the ESAS was an important tool for letting healthcare providers know how they feel [11]. However, no initiative to identify a common symptom screening tool has been undertaken in pediatric oncology.

It is important to distinguish between QoL instruments and symptom assessment scales as these are closely intertwined but distinct. QoL is a multidimensional construct grounded in the World Health Organization’s definition of health in which health is not merely the absence of disease, but rather, a state of complete physical, mental and social well-being [12]. Many QoL instruments include symptom assessment although their purpose is to measure the construct of QoL rather than the symptom specifically. In contrast, the purpose of symptom assessment scales is to identify and measure symptom burden.

In order to identify an optimal symptom screening tool that may be used in children receiving cancer treatment, it would first be important to describe all symptom assessment scales that have been used in this population. This process would allow one to determine if any of these scales may be used as a symptom screening tool or if one could be adapted for this purpose. Consequently, the objective was to describe symptom assessment scales that have been used in children receiving cancer treatment.

## Methods

### Data sources and searches

We used the Strengthening the Reporting of Observational Studies in Epidemiology (STROBE) guideline for reporting observational studies [13] to develop a protocol for this systematic review. We conducted electronic searches of OVID Medline (1948 to December 19, 2011) and EMBASE (1980 to December 19, 2011). Appendix 1 illustrates the search strategy.

### Study selection

We included studies that used a symptom assessment scale to measure multiple symptoms. Exclusion criteria were: (1) Not published as a full article (conference proceedings excluded); (2) Pediatric data not available; (3) Population not cancer; (4) Symptoms retrospectively reported for a period that did not include current symptoms (i.e. studies which used a recall period such as 1 week and 1 month were included while studies that only evaluated symptoms that occurred in the past and did not evaluate recent or current symptoms were excluded); (5) Purpose of the study was only to evaluate a translated version; (6) Not a study; (7) Duplicate publication; (8) Symptom assessment scale not appropriate because: a) only included psychological symptoms; b) included items that are not symptoms; or c) only measured a single symptom or (9) Not in English.

One reviewer (LS) evaluated the titles and abstracts identified by the search strategy and any potentially relevant publication was retrieved in full. Two independent reviewers (MCE and LS) assessed for eligibility. Final inclusion into the review was by agreement of both reviewers. Agreement between reviewers was evaluated using the kappa statistic. Strength of agreement as evaluated by the kappa statistic was defined as slight (0.00-0.20), fair (0.21-0.40), moderate (0.41-0.60), substantial (0.61-0.80) or almost perfect (0.81-1.00) [14].

### Data extraction, quality assessment and analytic approach

Two reviewers (MCE and LS) extracted data from included trials using a standardized data collection form. Data collected included trial demographics (year of publication, country in which study was conducted, language in which the instrument was administered), name of the instrument, information related to instrument administration (how administered, proxy or self-report, number of times administered), information about the number and characteristics of children enrolled (age, on/off active treatment) and the five most common symptoms identified. We also collected information about the purpose of instrument administration, whether treatment was altered as a result of this information and whether there were difficulties with administration for studies in which child self-report was used. We defined screening for this review as whether the study specifically reported abnormal results to clinicians or altered treatment because of identified symptoms. We then described the details of the identified scales.

Study quality was assessed using a modified version of an instrument previously developed to describe quality in studies of prognosis [15]. This instrument examines four potential sources of bias: study participation, study attrition, confounding variables and measurement of outcomes. Given that study attrition is less relevant in this setting, we excluded this item. Each element was rated as having low, medium or high risk of bias for each study.

The analytic approach was purely descriptive and the data were not synthesized.

## Results

**Figure 1:**
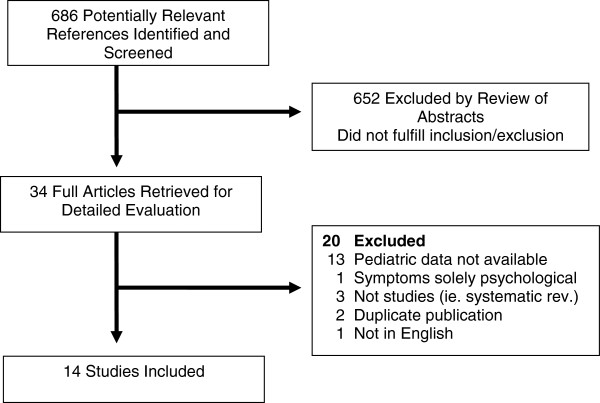
Flow diagram of study identification and selection.

Figure 1 illustrates the flow diagram of trial identification and selection. A total of 686 titles and abstracts were reviewed, and 34 full articles were retrieved. Of these, 14 satisfied pre-defined inclusion criteria. Reasons for excluding 20 articles are provided in Figure 1. The reviewers had perfect agreement on articles for inclusion (kappa = 1.00). The number of studies that illustrated low risk of bias was as follows: study participation (n = 6), confounding variables (n = 4) and measurement of outcomes (n = 7).

Table 1 illustrates the characteristics of the included studies. Of the 14 studies, [3,16-28] 6 were conducted in the United States[18-20,23,25,26] and 3 were conducted in the United Kingdom [16,24,28]. Twelve were conducted in English [3,16-20,23-28] and 2 were conducted in other languages in addition to English (Spanish [18] and Swedish [3]). Instruments were administered in person only (n = 10), by telephone only (n = 1) or in multiple formats (n = 3). None of the 14 studies used the symptom assessment scale to screen for symptoms and no study changed patient management on the basis of symptoms identified. The most common purpose of instrument administration was to describe the degree of symptom burden in their population (n = 8).

**Table 1:** Characteristics of studies that used a symptom assessment scale in pediatric oncology

**Author**	**Year**	**Instrument**	**Mean age in years ±SD or (range)**	**Number enrolled**	**Respondent**	Timing **	Once or multiple ***
Baggott[18]	2011	MSAS 10-18	14.7 ± 2.8	66	Child	During	Multiple
Wu[19]	2011	SDS	NS	40	Child	During	Once
Gibson[16]	2010	ASyMS-YG	15* (13–18)	27	Child	During	Multiple
Poder[3]	2010	MSAS 10-18	NS	292	Parent	During	Multiple
Walker[20]	2010	MSAS 7-12	14.2 + 2.7	51	Child	During	Multiple
Dupuis[17]	2009	Unique	9.4 ± 6.8	200	Parent	During	Once
Sitaresmi[21]	2009	Unique	6* (2–16)	61	Parent	During	Once
Yeh[22]	2009	MSAS 10-18	14.2 + 2.2	144	Parent	Both	Once
Williams[23]	2006	TRSC-C	10.4 ± 6.1	11	Parent	During	Once
Collins[24]	2002	MSAS 7-12	9.6 (7–12)	149	Child	Both	Multiple
Hinds[25]	2002	SDS	15.5 ± 2.1	77	Child	During	Multiple
Collins[26]	2000	MSAS 10-18	14 (10–18.2)	160	Child	Both	Multiple
Berard[27]	1998	RSCL	16.9	43	Child	During	Multiple
Eiser[28]	1997	RSCL	14.5 (4–19)	47	Parent	Both	Once

*Median; **Timing refers to whether instrument was administered during active treatment only or both during and following completion of chemotherapy; ***Instrument administered once or more than once.

Abbreviations: *ASyMS-YG* Advanced Symptom Management System – Young, *MSAS* Memorial Symptom Assessment Scale, *SDS* Symptom Distress Scale, *TRS-C* Therapy-related Symptom Checklist, *RSCL* Rotterdam Symptom Checklist, *SD* standard deviation, *NS* not stated.

Of the 11 studies that described the most common symptoms, the most frequently cited symptoms appearing on the 5 most common lists were: fatigue (n = 9), nausea (n = 7), pain (n = 5), drowsiness (n = 4), and anorexia (n = 3). There were 6 studies that described the mean number of symptoms per patient in their cohort; this number ranged from 1.9 to 12.7. Three of the studies which used child self-report noted that children sometimes needed assistance or clarification of questions [19,24,26].

Table 2 illustrates the details of the eight identified instruments including the number of items, description of items for scales that included < 15 items, dimensions and scale types.

**Table 2:** Features of the identified symptom assessment scales

**Instrument**	**Number of items**	**Items for those with < 15 Items**	**Dimensions**	**Type of scale**
ASyMS-YG[16]	5	Mouth sores, nausea, vomiting, weight loss, diarrhea	2: Severity and bother	3 or 4 point Likert-type scales
Dupuis[17]	69 and 71 depending on child age		3: Presence, severity and bother	5 point Likert-type scales
MSAS 7-12[24]	8	Lethargy, sadness, itchiness, pain, worry, anorexia, nausea, insomnia	3: Frequency, severity and distress	3 or 4 point Likert-type scales
MSAS 10-18[26]	30		3: Frequency, severity and distress	4 or 5 point Likert-type scales
RSCL[29]	39		1: Bother	Mainly 4 point Likert-type scales
SDS[25]	10	Sleep, feeling, tiredness, appearance, appetite, ability to get around pain, nausea, bowel movements, concentration	1: Distress	5 point Likert-type scales
Sitaresmi[21]	13	Nausea, vomiting, abdominal pain, mouth ulcers, increased appetite, decreased appetite, infections, excessive weight gain, hair loss, leg weakness, fatigue, spontaneous bleeding, behavior alteration	2: Frequency and severity	5 point Likert-type scales
TRSC-C[23]	23		1: Severity	5 point Likert-type scales

Abbreviations: *ASyMS-YG* Advanced Symptom Management System – Young; *MSAS* Memorial Symptom Assessment Scale, *RSCL* Rotterdam Symptom Checklist, *SDS* Symptom Distress Scale, *TRSC-C* Therapy-related Symptom Checklist – Child.

## Discussion

We identified 14 studies that used eight different symptom assessment scales to measure symptoms in children with cancer. The most common use of these scales was to describe the prevalence of symptom burden. None were used as a symptom screening tool and none were used to influence patient management. Consequently, there is an absence of symptom screening tools which have been used in children with cancer.

Measuring symptom severity in children is critical. Children undergoing cancer treatment suffer and may only seek help when symptoms become severe [16,30]. In one study in which children 13–18 years of age completed an electronic version of a symptom questionnaire, participants noted that self-reporting symptoms was reassuring, made them feel more in control, helped them to remember their symptoms and allowed them to see how symptoms changed over time [16].

Identifying a feasible and clinically useful symptom screening tool is important. Symptom screening instruments could be used by patients in routine clinical practice in order to identify problems and focus the families’ and healthcare providers’ attentions on symptom control. These instruments may also be used to determine symptom prevalence and thereby inform the prioritization of clinical patient services and/or research resources. In considering an ideal screening instrument, the scope of symptoms should include the most important symptoms to the patient. The instrument should take into account the perspective of the patient’s family regarding symptom impact, be applicable to children of all ages and have adequate psychometric properties such as reliability and validity. Both parent-proxy versions and child self-report versions would be important to address the needs of children of different ages and cognitive abilities. In order to be feasible in clinical practice, a brief screening tool is likely to be more successful than lengthy assessment scales.

Once a feasible and clinically useful screening tool is identified for pediatric cancer, a future step could be to identify, adapt or develop evidence-based guidelines for the management of each symptom included in the tool. Such a system could improve patient/family self-management and improve the ability of healthcare professionals to standardize monitoring and care.

Our study has important limitations. First, we only included studies published in the English language. The rationale for this decision is that our research plan is to first identify or adapt a symptom screening tool for use in English with later translation into other languages. Second, it is possible that there are symptom screening tools being used in practice that have not been evaluated in the peer-reviewed literature. Another limitation of our study is the exclusion of scales which address psychosocial symptoms alone. A final limitation is that our review excluded single symptom scales. Although these scales are extremely important in clinical practice and research, they do not address our goal of identifying a scale which could be used as a symptom screening instrument or adapted for this purpose. A future goal will be to examine the eight symptom assessment scales identified in this review and determine if one of these could be used as a symptom screening tool or if one could be adapted for this purpose. Such a goal would likely be best accomplished using a consensus methodology among a multi-disciplinary group of experts in pediatric oncology supportive care.

## Conclusion

In conclusion, we performed a systematic review of symptom assessment scales and identified eight instruments which have been used in children with cancer; none were used for the purpose of screening of symptoms or altered care. Identification or development of a symptom screening tool in pediatric oncology should be a priority.

## Appendix 1

Details of the Literature Searches.

### Ovid MEDLINE(R) 1948 to Present

1. (Symptom* adj2 (Scale* or instrument* or screen* or measure* or tool or tools)).mp. (9775)

2. exp neoplasms/ (2334147)

3. 1 and 2 (1100)

4. ((edmonton or Memorial) adj2 Symptom* adj2 Assess* adj2 (Scale* or instrument* or screen*)).mp. (211)

5. 3 and 4 (1151)

6. limit 5 to "all child (0 to 18 years)" (120)

### EMBASE <1980 to 2011 Week 50>

1. (Symptom* adj2 (Scale* or instrument* or screen* or measure* or tool or tools or checklist*)).mp. or symptom checklist 90/ or symptom distress scale/ or brief symptom inventory/ or ((edmonton or Memorial) adj2 Symptom* adj2 Assess* adj2 (Scale* or instrument* or screen*)).mp. (21462)

2. physical disease by body function/ or constipation/ or exp coughing/ or exp cyanosis/ or exp diarrhea/ or exp faintness/ or exp fatigue/ or exp incontinence/ or listlessness/ or malaise/ or exp "nausea and vomiting"/ or exp pain/ or pallor/ or weakness/ (954957)

3. checklist/ or clinical assessment tool/ or rating scale/ or scoring system/ or exp screening/ or summated rating scale/ or self report/ (572563)

4. 1 or (2 and 3) (68751)

5. exp neoplasm/ or cancer patient/ or exp terminally ill patient/ (2708653)

6. 4 and 5 (9312)

7. limit 6 to (infant < to one year > or child < unspecified age > or preschool child <1 to 6 years > or school child <7 to 12 years > or adolescent <13 to 17 years>) (628)

8. 2 or 4 (2)

9. 11 or 12 (628)

## Abbreviations

ESAS: Edmonton Symptom Assessment Scale; QoL: Quality of life.

## Competing interests

The authors declare that they have no competing interests.

## Authors’ contributions

LLD, DT, MCE, TH and LS have made substantial contributions to study conception and design, acquisition of data and analysis and interpretation of data. All have been involved in drafting and critical revision of the manuscript for important intellectual content and have given final approval of the version to be published.

## Pre-publication history

The pre-publication history for this paper can be accessed here:

http://www.biomedcentral.com/1471-2407/12/430/prepub
